# Creating multifaceted allied health professionals with skilled hands and the power of words

**DOI:** 10.3389/fmed.2025.1673811

**Published:** 2025-11-10

**Authors:** Urmila Kagal, Sharmila Jalgaonkar, Pankaj Gupta, Nayana Hashilkar

**Affiliations:** 1 Department of Pharmacology, J. N. Medical College, KLE Academy of Higher Education and Research, Deemed-to-Be-University, Belagavi, India; 2 Department of Pharmacology, Seth GS Medical College and KEM Hospital, Mumbai, India; 3 Department of Conservative Dentistry and Endodontics, Nair Hospital Dental College, Mumbai, India

**Keywords:** allied health, communication, Healthcare, skills, professionals

## Abstract

**Background:**

Allied health professionals (AHPs) are a crucial cog in the wheel of the healthcare industry. AHPs contribute immensely to public health and wellbeing. To create employable AHPs, we must focus on a multi-faceted approach which empowers them not merely with technical expertise, but the ability to articulate themselves clearly. The present study was planned to assess the effectiveness of patient centered communication skill training in improving communication skills in final year undergraduate allied health students using a modified version of The Kalamazoo Essential Elements Communication Checklist (KEECC). It also aimed to assess the effectiveness of patient centered communication skill training in improving patient satisfaction using a modified version of The Patient Satisfaction Questionnaire 18 (PSQ) Short Form.

**Methods:**

The present study was an educational study using the pre post study design. The total duration of the study was 6 months. Universal sampling was followed. Hence, all students admitted to various allied health programs in the academic year 2022–23, suitable as per the inclusion criteria were recruited, which resulted in 65 students being enrolled in the study. A communication skills training module was used to train students in patient centered communication. Students were assessed before and after training by faculty and patients using The Kalamazoo Essential Elements Communication Checklist (KEECC) and The Patient Satisfaction Questionnaire (PSQ) Short Form, respectively.

**Results:**

Significant improvement in communication skills and patient satisfaction was found after exposure to the training module as indicated by significant changes in KEECC and PSQ scores, respectively after applying the paired *t*-test. *P*-value of <0.05 was considered significant.

**Conclusion:**

The present study proves that enhancing patient focused communication of the allied health professionals who are healthcare’s work horses with skilled hands, improves patient satisfaction. Policy makers of public health and health science institutions should take cognizance of the fact that, empowering these professionals through a multifaceted approach involving honing their technical acumen, patient centered communication and teamwork would help in creating a workforce which can meet the demands of contemporary healthcare and have a positive impact on public health.

## 1 Introduction

Allied Health professionals (AHPs) have a pivotal role to play in the delivery of health services, pertaining to the identification, assessment, and prevention of diseases. In addition, they provide nutrition services, rehabilitative services and assist in managing of health systems (1). An “allied health professional” includes an associate, technician or technologist who is trained to perform any technical and practical task to support diagnosis and treatment of illness, disease, injury or impairment. This group of healthcare professionals through their enthusiasm, expertise and opportunity contribute to disease prevention and health improvement and hence have a crucial role to play in public health and wellbeing (2, 3). If the skills of AHPs are used effectively across the system, local communities and individuals can benefit from their skills, knowledge and expertise which can lead to a positive change in the health, social and wider care system (4).

Allied health professionals as a vital part of the healthcare team are required to communicate with doctors, nurses and medical residents as a part of collaborative health practice at case conferences, team meetings, hallway and staff room discussions. “Effective communication” has been recognized as a core non-technical skill and a means of providing knowledge, building relationships and effective team coordination. Efficient teamwork results in delivery of high-quality healthcare and better patient safety. “Communication failures” have long been regarded as the principal cause of unintentional patient harm (5). In modern practice of healthcare, people from a range of healthcare professions are required to work together across varied contexts and hence, it has become increasingly important to create a workforce which can meet the demands of collaborative practice which is in keeping with the pre-requisites of contemporary healthcare (6).

In India and the Global South, the immense contribution of the allied health professionals to healthcare industry has been undermined and so has been the teaching and training of this category of students. It is these individuals who spend more time with patients than do physicians. They also remain involved in a patient’s care throughout the duration of the patient’s stay in the hospital (7).

In India we finally see some light at the end of the tunnel following the drafting of new competency- based curricula for various allied health programs with communication skill training as a part of the curriculum. These curricula have been drafted by the National Commission for Allied and Healthcare Professions (NCAHP), Government of India under the NCAHP Act, 2021. This has been added with the intention of training allied health students to communicate effectively with patients/clients, care- givers, other health professionals, and other members of the community effectively and appropriately. These curricula will be applicable from the academic year 2026–27. But only time will tell whether this will be successfully implemented all over the country (8).

### 1.2 Objectives of the study

To assess the effectiveness of patient centered communication skill training in improving communication skills in final year undergraduate allied health students using a modified version of The Kalamazoo Essential Elements Communication Checklist (KEECC).

To assess the effectiveness of patient centered communication skill training in improving patient satisfaction using a modified version of The Patient Satisfaction Questionnaire 18 (PSQ) Short Form.

## 2 Materials and methods

### 2.1 Study design and selection of study participants

The present study was an educational study with a pre post intervention study where the intervention used was a training module on patient centered communication skills. The total duration of the study was 6 months. The study was approved by the Ethics Committee of the institution as per reference number MDC/JNMCEC/504 dated 30th July 2024. The study was conducted in accordance with the norms of the Declaration of Helsinki.

Inclusion Criteria:

Final year undergraduate students enrolled in various allied health programs run by the university who voluntarily consented to participate in the study were recruited after seeking written informed consent. The students belonged to the following allied health programs:

B.Sc. Medical Laboratory Technology

B.Sc. Cardiac Care Technology

B.Sc. Optometry

B.Sc. Radiography

Exclusion Criteria:

1. Students who refused to participate in the study.
2. Students enrolled in programs where they dealt mainly with patients who were in altered consciousness such as critical care technology, anesthesia technology and perfusion technology.
3. Students who remained absent for the baseline assessment of communication skills before exposure to the training module.
4. Students who remained absent during the delivery of the training module.
5. Students who remained absent for the assessment of communication skills after exposure to the training module.

### 2.2 Study sample

#### 2.2.1 Sampling method: universal sampling

##### 2.2.1.1 Sample size

All students admitted to various allied health programs suitable for enrollment in the study as per the inclusion criteria admitted to the educational institution in the academic year 2022–23 were recruited in the study only after they agreed to voluntarily participate by providing written informed consent. Hence, 65 students who were in their final year of under graduation were recruited in the study.

### 2.3 Data collection

Attitude of the recruited students toward communication skills was assessed before exposure to the communication skill training module by using a modified version of the Communication Skills Attitude Scale (CSAS) (9). Students were asked to communicate with real world patients and were assessed for effectiveness of communication skills by faculty before and after training using a modified version of the Kalamazoo Essential Elements Communication Checklist - KEECC (10). Patient satisfaction before and after training was assessed by patients using a modified version of Patient Satisfaction Questionnaire - Short Form (PSQ 18) (11).

The modified versions of CSAS, KEECC and PSQ 18 were validated for their content by medical education experts before they were used for the study. Each item/statement in each of the checklists viz CSAS, KEECC and PSQ 18 was followed by five boxes in a consecutive order on a Likert scale, named “Strongly disagree,” “Disagree,” “Uncertain,” “Agree” and “Strongly agree” and was scored from 1 to 5, respectively. Some of the items in these questionnaires are positively worded and negatively worded. The student/assessor/patient was instructed to tick one box only. Before analyzing the data, the scores were reversed for the negative items to obtain the same direction of scores for both negative and positive items i.e., a higher score represents more positive attitudes for all items in each of these questionnaires (12).

#### 2.3.1 Quantification of content validity

Kappa statistic is a consensus index of inter-rater agreement that adjusts for chance agreement. It serves as an important supplement to content validity index (CVI) because Kappa provides information about the degree of agreement beyond chance. To calculate the modified Kappa statistic, the probability of chance agreement is first calculated for each item by the following formula:

PC = [N! / A! (N − A)!] × 0.5^N^

In this formula, N = number of experts in a panel and A = number of panelists who agree that the item is relevant. After calculating I-CVI for all instrument items, finally, Kappa was computed by entering the numerical values of probability of chance agreement (PC) and CVI of each item (I-CVI) in the following formula: K = (I-CVI − PC) / (1 − PC).

I-content validity index is calculated as the number of experts giving a rating 3 or 4 to the relevancy of each item, divided by the total number of experts. The I-CVI expresses the proportion of agreement on the relevancy of each item, which is between 0 and 1.

Evaluation criteria for Kappa are the values above 0.74, between 0.60 and 0.74, and the ones between 0.40 and 0.59 are considered as excellent, good, and fair, respectively (13–15).

The content validation was performed a panel of 10 experts. Following this, the formulae mentioned above were used and the Kappa values were found to be the following:

For the modified CSAS, each item yielded a Kappa value of 1.

For the modified KEECC, each item yielded a Kappa value of 1 except items 4 and 6 which yielded a Kappa value of 0.89.

For the modified PSQ 18 each item yielded a Kappa value of 1.

#### 2.3.2 Following are the details of the training module

##### 2.3.2.1 Duration of the training module

The students were exposed to two lecture sessions on two consecutive days. Each lecture lasted for 2 h. Following the lecture sessions on the third day, there were two role plays each of a half hour duration performed by the faculty members.

##### 2.3.2.2 Teaching methods

The teaching method involved using interactive lectures which were covered under the following headings:

(1) Building the technicians-patient relationship.

(2) Principles of good communication consisting briefly of: opening the discussion, gathering information, demonstrating empathy, brief explanation of the procedure which will be performed on the diagnostic / therapeutic procedure which will be performed on the patient after obtaining informed consent.

(3) Summary / Basic layout of patient centered communication.

The lectures were delivered by faculty trained in medical education using a power point presentation. The lectures were made interactive to break the monotony by using methods such as think, pair and share and buzz groups.

##### 2.3.2.3 Interactive methods used during lectures

During the first lecture we used think, pair and share.

During the second lecture, we used brain storming.

Details are given below.

Think: The teacher would pose an open-ended question or problem, and students were given a short period of time to think about it individually.

Pair: Students formed pairs with the classmate sitting beside them and discussed their ideas, answers, and perspectives with each other.

Share: Some pairs were called upon to share their discussion with the whole class, leading to a broader discussion and varied perspectives.

Buzz group: Small discussion groups were formed within the larger group consisting of six students, formed to brainstorm, solve problems, or share ideas on a specific small topic within a short timeframe. After the short discussion within the small group, a spokesperson from each buzz group typically reports the group’s key points to the larger assembly.

##### 2.3.2.4 Details of role play

The role plays mimicked a real-world situation with one faculty member posing as a patient, another posing as the patient’s attendant and another as a technician. One role play demonstrated good patient centered communication and another demonstrated poor communication skills.

### 2.4 Quantitative analysis

Five students were excluded from the final analysis since two of them were absent during delivery of the communication skills training module and three of them were absent during assessment following delivery of the module.

Median score was calculated for every item on CSAS. The rest of the data was expressed as mean ± standard deviation (SD). The differences in the mean scores obtained on every item of the KEECC as well as PSQ 18 before and after conduct of the training session were analyzed by the paired *t*-test. *P*-value of <0.05 was considered significant. Data was analyzed using Graph Pad Prism version 10.4.1.

## 3 Results

### 3.1 Attitude toward communication skills

The median scores were higher for various negative attitude statements (NAS) and lower for various positive attitude statements (PAS) on modified CSAS as shown in Table 1. PAS have been indicated in the table by italics. The total of the median scores was 46 and 26 for NAS and PAS, respectively.

**TABLE 1:** Median scores of items on the modified Communication Skills Attitude Scale (CSAS).

S. No.	CSAS item	Median
1	In order to be a good AHP, I must have good communication skills	2.5
2	I can’t see the point in learning communication skills	4.5
3	Nobody is going to fail in their under graduate degree exam for having poor communication skills	5
4	Developing my communication skills is just as important as developing my knowledge of technical skills	2
5	Learning communication skills has helped or will help me respect patients	2.5
6	I haven’t got time to learn communication skills	3.5
7	Learning communication skills is interesting	2
	I can’t be bothered to turn up to sessions on communication skills	3.5
9	Learning communication skills has helped or will help facilitate my team working skills	2
10	Learning communication skills will improve my ability to communicate with patients	2.5
11	Communication skills teaching states the obvious and then complicates it	3.5
12	Learning communication skills is fun	1.5
13	Learning communication skills is too easy	4.5
14	Learning communication skills has helped or will help me respect my colleagues	2.5
15	Learning communication skills has helped or will help me recognize patients’ rights regarding confidentiality and informed consent	2.5
16	Communication skills teaching would have a better image if it sounded more like a science subject	4.5
17	I don’t need good communication skills to be an AHP	4.5
1	I find it hard to admit having some problems with my communication skills	3.5
19	I think it’s really useful learning communication skills on the AHP degree	2
20	My ability to pass exams will get me through under graduation rather than my ability to communicate	4.5
21	Learning communication skills is applicable to learning allied health sciences	2
22	Learning communication skills is important because my ability to communicate is a life–long skill	2
23	Communication skills learning should be left to psychology students, not AHP students	
	PAS score	26
	NAS score	46

### 3.2 Assessment of communication skills

Higher scores were found in various communication skill competencies in the modified KEECC checklist after delivery of the training module as indicated by significant *p*-values after applying the paired *t*-test as shown in Table 2.

**TABLE 2:** Effect of communication skill training of allied health students on the modified Kalamazoo Essential Elements Communication Checklist (KEECC).

Communication skill competency	Score before training (mean ± SD)	Score after training (mean ± SD)	t statistic	*P*-value
**Build a relationship**				
Greets and shows interest in patient as a person	2.3 ± 1.09	3.7 ± 0.46	11. 86	<0.0001
Uses words that show care and concern throughout the interview	2.3 ± 1.09	3.5 ± 0.49	10.6	<0.0001
Uses tone, pace, eye contact, and posture that show care and concern	2.2 ± 1.07	3.6 ± 0.49	12.67	<0.0001
**Open the discussion**				
Allows patient to complete opening statement without interruption	2 ± 1.15	3.5 ± 0.62	12.62	<0.0001
Asks “Is there anything else?” to elicit full set of concerns	2.2 ± 1.11	3.5 ± 0.62	11. 85	<0.0001
**Gather information**				
Begins with patient’s story using open-ended questions (“Tell me about …”)	2.1 ± 1.07	3.4 ± 0.69	12.45	<0.0001
Clarifies details as necessary with more specific or “yes/no” questions	2.1 ± 1.07	3.7 ± 0.54	12.52	<0.0001
**Understand the patient’s perspective**				
Responds explicitly to patient statements about ideas, feelings, and values	1.8 ± 0.66	2.2 ± 0.54	6.05	<0.0001
**Share information**				
Explains using words that are easy for patient to understand	2.2 ± 1.09	4.2 ± 0.49	13. 81	<0.0001
Asks whether patient has any questions	1.7 ± 0.83	4.2 ± 0.49	19.93	<0.0001
**Provide closure**				
Asks whether the patient has questions, concerns, or other issues	1.7 ± 0.83	4.2 ± 0.49	19.93	<0.0001
Summarizes	2 ± 0.93	4 ± 0.51	15.27	<0.0001
Acknowledges patient and closes interview	2.2 ± 0.84	4.1 ± 0.43	17.08	<0.0001

### 3.3 Assessment of patient satisfaction

Higher scores were found in various items in the modified PSQ short form checklist after delivery of the training module as indicated by significant *p*-values after applying the paired *t*-test as shown in the Table 3. The patient satisfaction with respect to technical competence of the students did not change significantly.

**TABLE 3:** Effect of communication skill training of allied health students on the modified Patient Satisfaction Questionnaire (PSQ) short form.

PSQ item	Satisfaction score before training	Satisfaction score after training	t statistic	*P*-value
**General satisfaction**				
The care I have been receiving from the technician is just about perfect.	2.6 ± 0.95	3.9 ± 0.51	9.91	<0.0001
I am dissatisfied with some things about the care I receive.	2.5 ± 0.87	3.9 ± 0.48	13. 81	<0.0001
**Technical quality**				
I think my technician has the technical expertise to provide the necessary diagnostic / therapeutic services	4.1 ± 0.91	4.1 ± 0.87	13	0.0832
Sometimes technicians make me wonder whether their technical acumen is adequate	3.4 ± 0.78	3.4 ± 0.76	14.07	0.1819
When I go for a procedure, they are careful to ensure that the necessary devices are in working condition	3.3 ± 0.64	3.4 ± 0.66	13.94	0.013
I have some doubts about the technical ability of the technicians who attend to me	3.5 ± 0.78	3.6 ± 0.76	10.5	0.1590
**Interpersonal manner**				
Technicians act too business like and impersonal toward me	1.9 ± 1.04	3.8 ± 0.57	1.76	<0.0001
Technicians treat me in a very friendly and courteous manner.	1.9 ± 1.03	3.7 ± 0.6	1.35	<0.0001
**Communication**				
Technicians are good about explaining the reason for medical tests.	2.4 ± 0.89	3.8 ± 0.54	2.56	<0.0001
Technicians sometimes ignore what I tell them	2.4 ± 0.86	3.6 ± 0.49	1.42	<0.0001
**Time spent with technician**				
Those who provide technical services sometimes hurry too much when they treat me.	2.7 ± 0.72	3.8 ± 0.36	12.07	<0.0001
Technicians usually spend plenty of time with me.	3 ± 0.67	4 ± 0.53	25.61	<0.0001

## 4 Discussion

The present study was planned with the objective of assessing how effective patient centered communication skill training was, when offered to final year undergraduate allied health students would be in improving communication skills and patient satisfaction. The data of 60 students was included in the analysis.

Students showed a negative attitude toward communication skills as indicated by the higher median Negative attitude statement (NAS) scores as compared to positive attitude statements (PAS) scores on the CSAS. Significant improvement in communication skills was found after exposure to the training module as indicated by significant changes in KEECC and PSQ 18 scores.

Communication forms the very foundation of the healthcare industry through interaction between the healthcare consumers and providers and between the providers themselves as a part of an efficient healthcare delivery team (16).

Good communication involves not only the ability to communicate clearly and completely, but also respecting others and listening intently to what they have to say. Miscommunications can have serious repercussions. A lapse in communication can result in delayed reports, wrong interpretations, or even risks to patient safety. Resolution of conflicts occurs when everyone behaves in a professional and respectful manner. Those who want to achieve professional growth will always remain conscious of practicing good communication skills, and will always be receptive to feedback, to achieve self-improvement (17).

In the present study the final year undergraduate allied health students had a negative attitude toward learning communication skills. They had never thought of effective communication as a skill which needs to be acquired through formal training. It had never dawned on them that effective communication is as crucial as technical expertise and that the two are complementary to each other. There were no similar studies in the literature on the attitude of the allied health students toward learning communication skills. Also, there were no studies involving use of KEECC to determine the effectiveness of a communication skill training module on allied health students. Hence, the present study was compared with studies performed on medical undergraduates.

In contrast to our study, Nayak et al. found that the attitude of medical undergraduate students in the 3rd year of under graduation had a positive attitude toward communication skills. Attitude toward learning communication skill was assessed using the CSAS. The CSAS median score for positive attitude was 57.5 and for negative attitude was 25 (minimum score = 13 and maximum score = 65) (18).

In the present study, the negative attitude of the students was reflected by the observation that, they had poor communication skills before they were exposed to the training module. It was found that the students lacked even basic courtesies such as greeting the patient, showing concern, and opening the conversation. The student would straight away get down to performing the procedure under the guidance of a senior technician but without bothering to explain the details of the procedure. This has been observed through the poor scores of the students as per the communication competencies mentioned in the KEECC. Following training there was a significant improvement in the KEECC scores implying that the training module was effective in delivery of the required communication skills to the AHP students.

The negative attitude of the students in the present study was also reflected by the observation that patient satisfaction scores were poor before students were exposed to the training module. There was a significant improvement in patient satisfaction after training as indicated by significant improvement in the PSQ scores after training.

Kalia et al. conducted a study to evaluate a communication training program and perceptions of medical interns and faculty toward it. Sixty medical interns were enrolled for communication skill training which was later assessed using the Kalamazoo scale. Attitude of the interns toward the same was assessed using Rees and Sheard Scale. Attitude of faculty toward introduction of communication skill training was also assessed. In the present study, the mean score of PAS and NAS was 59.15 ± 5.51 and 25.20 ± 9.60, respectively. All the faculty members believed communication skill training should be a part of the curriculum. Majority (75%) opined that it should be introduced right from the beginning while 25% wanted it before clinical postings (19).

Mata ÁNS et al. performed a systematic review for which they retrieved eight studies that addressed training programs in communication skills for the health professional and patient relationship. Improvements were observed in the performance and self-efficacy of professionals with regard to communication skills. These skills were taught through different teaching strategies, involving experiential activities, which are fundamental for the improvement of care and for patient-centered attention (20).

Nguyen et al. conducted a study on 40 optometry students where in the students were expected to interact with a volunteer patient through an online teleconferencing platform which was observed by a teaching clinician. Patients and clinicians evaluated the interpersonal skills of the student using qualitative written feedback, and a quantitative rating (Doctors’ Interpersonal Skills Questionnaire). All participants were requested to complete an anonymous survey at the completion of the study. The study concluded that multisource feedback about interpersonal skills contributes to improvement in student performance (21).

The Patient Advisory Group (PAG) of the European Society of Radiology, in collaboration with the European Federation of Radiographer Societies (EFRS) highlights the important role that communication plays when trying to meet patients’ expectations through their entire imaging journey in the radiology department. According to the experts, radiography teams should be frequently reviewing their existing protocols to ensure that they support the opportunity for good communication with patients. The key areas of consideration have been summarized in an easy-to-remember mnemonic: COMMUNICATION- Compassionate care, Organized, Medical terms, make a difference, Understanding, Notice, Informed, Consent, Assurance, Trust, Interpersonal communication, Openness and Needs. This mnemonic serves as a reminder to radiographers to work toward improving patient-radiographer interactions and provide patient-focused services (22).

A study was conducted by Meenakshi Rani to explore how medical laboratory technicians (MLTs), MLT interns, and MLT students perceive and practice communication in their daily work. The study concluded that in addition to technical skills, communication skills are absolutely necessary to ensure that technical skills translate into safe and effective patient care. This study highlights the growing need to equip MLTs with strong interpersonal skills and embedding communication training into the MLT curriculum as a compulsory course. This would improve efficiency of not just delivery of laboratory services but would also strengthen the overall healthcare system (23).

The present study found that there was no significant difference in patient satisfaction with respect to the technical skills of these students after exposure to the training module in communication skills. This is because these students were already proficient technically. These students were in the final year of under graduation and had already gained technical expertise because of regular teaching and training offered by the faculty with respect to technical skills.

### 4.1 Strengths of the study

This study was first study of it’s kind in the institution. Also, very few similar studies were available in the literature on allied health professionals for comparison with the present study. This study was conducted in a real world setting and hence, provided the students with an opportunity for experiential learning. The process of learning was made interesting by using student centric learning methods such as think pair share, buzz groups and role plays.

### 4.2 Limitations of the study

This study only tested the effect of communication skill training on the immediate learning outcome. Assessment of long-term learning has not been tested. Students who deal mainly with patients who were in altered consciousness such as critical care technology, anesthesia technology and perfusion technology could not be included in the study. A training module tailor made to the requirement of such students needs to be created and tested.

## 5 Conclusion

The present study proves that enhancing patient focused communication of the allied health professionals who are healthcare’s work horses with skilled hands, improves patient satisfaction. Policy makers of public health and health science institutions should take cognizance of the fact that, empowering these professionals through a multifaceted approach involving honing their technical acumen, patient centered communication and teamwork would help in creating a workforce which can meet the demands of contemporary healthcare and have a positive impact on public health.

## Data Availability

The original contributions presented in this study are included in this article/supplementary material, further inquiries can be directed to the corresponding author.
